# Author’s Reply: "Data Contamination in AI Evaluation"

**DOI:** 10.2196/82057

**Published:** 2025-09-29

**Authors:** ChulHyoung Park, Min Ho An, Gyubeom Hwang, Rae Woong Park, Juho An

**Affiliations:** 1 Department of Biomedical Informatics School of Medicine Ajou University Suwon Republic of Korea; 2 Center for Biomedical Informatics Research Ajou University Medical Center Suwon Republic of Korea; 3 Department of Medical Sciences Graduate School of Ajou University Suwon Republic of Korea; 4 BK21 R&E Initiative for Advanced Precision Medicine Suwon Republic of Korea; 5 Department of Emergency Medicine School of Medicine Ajou University Suwon Republic of Korea

**Keywords:** artificial intelligence, large language model, ChatGPT, emergency medicine, clinical performance examination, history taking, clinical reasoning, empathy, patient experience

We sincerely thank the author for the constructive commentary on our recent publication. Our study evaluated ChatGPT’s performance across multiple dimensions—including history taking, diagnostic accuracy, communication skills, and empathic expression—through a clinical performance examination using simulated patients combined with written examinations [1].

In our study, the written examination was not intended to solely serve as a direct comparison of performance between ChatGPT and human physicians. Rather, it was included to support the interpretation of ChatGPT’s communication skills and empathic responses observed during simulated patient interactions by providing additional context regarding the model’s underlying clinical knowledge. A previous study has shown that patients may perceive ChatGPT’s responses as empathic or trustworthy, even when those responses are clinically inappropriate [2]. However, effective clinical communication is not merely about verbal fluency or emotional tone; it must be grounded in adequate medical knowledge. For this reason, earlier studies evaluating artificial intelligence empathy have also assessed the clinical appropriateness of responses and compared them to those of human physicians [2,3].

Consistent with prior work, we also assessed the simulated patient conversations in terms of both clinical accuracy and empathic engagement, as evaluated by an emergency medicine professor. However, we recognize that physicians vary in their diagnostic styles and communication approaches. Subjective judgment from the evaluator may have influenced the ratings, especially given that the evaluated outputs were full conversations rather than single responses. To provide a complementary and more structured assessment, we incorporated a written test focused on 3 key domains: diagnosis, investigation, and treatment planning. Performance on this test may serve as a supporting element to help ensure that ChatGPT’s interpersonal strengths were not misaligned with clinical reasoning.

As the author correctly pointed out, the questions in the written examination were adapted from a publicly available textbook published in 2018 [4]. We cannot rule out the possibility that ChatGPT was exposed to this material or similar content during pretraining, due to the limited transparency regarding its training data. Therefore, part of the model’s performance on the written test may have been influenced by data contamination. We fully acknowledge this methodological limitation and agree that the results from the written examination should be interpreted with caution.

We are truly grateful for the author’s thoughtful engagement, which raises important considerations for future studies regarding the assessment of AI in clinical settings.
